# Carrier properties of B atomic-layer-doped Si films grown by ECR Ar plasma-enhanced CVD without substrate heating

**DOI:** 10.1080/14686996.2017.1312520

**Published:** 2017-04-27

**Authors:** Masao Sakuraba, Katsutoshi Sugawara, Takayuki Nosaka, Hisanao Akima, Shigeo Sato

**Affiliations:** ^a^ Laboratory for Nanoelectronics and Spintronics, Research Institute of Electrical Communication, Tohoku University, Sendai, Japan

**Keywords:** Epitaxial growth, atomic-layer doping, plasma-enhanced chemical-vapor deposition, argon, silicon, boron, carrier mobility, Hall effect, 40 Optical, magnetic and electronic device materials, 105 Low-Dimension (1D/2D) materials, 201 Electronics / Semiconductor / TCOs, 212 Surface and interfaces, 302 Crystallization / Heat treatment / Crystal growth, 305 Plasma / Laser processing, 306 Thin film / Coatings

## Abstract

The atomic-layer (AL) doping technique in epitaxy has attracted attention as a low-resistive ultrathin semiconductor film as well as a two-dimensional (2-D) carrier transport system. In this paper, we report carrier properties for B AL-doped Si films with suppressed thermal diffusion. B AL-doped Si films were formed on Si(100) by B AL formation followed by Si cap layer deposition in low-energy Ar plasma-enhanced chemical-vapor deposition without substrate heating. After fabrication of Hall-effect devices with the B AL-doped Si films on unstrained and 0.8%-tensile-strained Si(100)-on-insulator substrates (maximum process temperature 350°C), carrier properties were electrically measured at room temperature. Typically for the initial B amount of 2 × 10^14^ cm^−2^ and 7 × 10^14^ cm^−2^, B concentration depth profiles showed a clear decay slope as steep as 1.3 nm/decade. Dominant carrier was a hole and the maximum sheet carrier densities as high as 4 × 10^13^ cm^−2^ and 2 × 10^13^ cm^−2^ (electrical activity ratio of about 7% and 3.5%) were measured respectively for the unstrained and 0.8%-tensile-strained Si with Hall mobility around 10–13 cm^2^ V^−1^ s^−1^. Moreover, mobility degradation was not observed even when sheet carrier density was increased by heat treatment at 500–700 °C. There is a possibility that the local carrier (ionized B atom) concentration around the B AL in Si reaches around 10^21^ cm^−3^ and 2-D impurity-band formation with strong Coulomb interaction is expected. The behavior of carrier properties for heat treatment at 500–700 °C implies that thermal diffusion causes broadening of the B AL in Si and decrease of local B concentration.

## Graphical abstract

**Figure:**
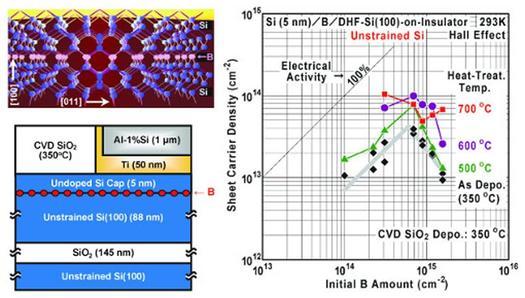

## 1. Introduction

Development of semiconductor devices with lower power consumption is increasingly required for Si large-scale integrated circuits (Si LSI). For that purpose, quantum-tunneling devices, such as Esaki-tunnel field-effect transistors [1–4] and resonant-tunneling devices [5–7], are now being developed. The key technology is energy-band modulation using heterostructures and local doping [8]. Especially for high-performance group-IV semiconductor devices, wide-range and precise control of local impurity concentration is required to generate carriers and ionized atoms, and lower-temperature epitaxy processes have been pursued because suppression of intermixing at interfaces is indispensable [9–15].

Plasma-enhanced chemical-vapor deposition (CVD) is expected to be useful to lower the deposition temperature and to obtain heavily doped films out of thermal equilibrium with suppressed thermal diffusion. Even at lower temperatures below 400 °C, by utilizing the plasma-enhanced CVD, Si epitaxial growth on Si has been demonstrated, to develop a lower-thermal-budget process for advanced solar cells, metal-oxide-semiconductor field-effect-transistors, etc [16–20]. Furthermore, our previous works demonstrated an electron-cyclotron-resonance (ECR) Ar plasma-enhanced CVD process as a candidate for low-temperature CVD, and epitaxial growth of strained/unstrained films of Si, Ge, Si-Ge alloy and Si-C alloy and *in situ* doping in Si and Ge have been achieved without substrate heating [21–30]. Here, the use of relatively low-energy plasma is expected to simplify the epitaxial growth process by eliminating substrate heating, and this might suppress redundant phenomena such as reaction of physically adsorbed contaminants on hydrogen-terminated Si surfaces, outgassing and various temperature-related instabilities. For advanced electronic device applications, the precise control of heterostructure formation and *in situ* doping in the ECR Ar plasma-enhanced CVD are now being investigated [25,29].

**Figure 1.**
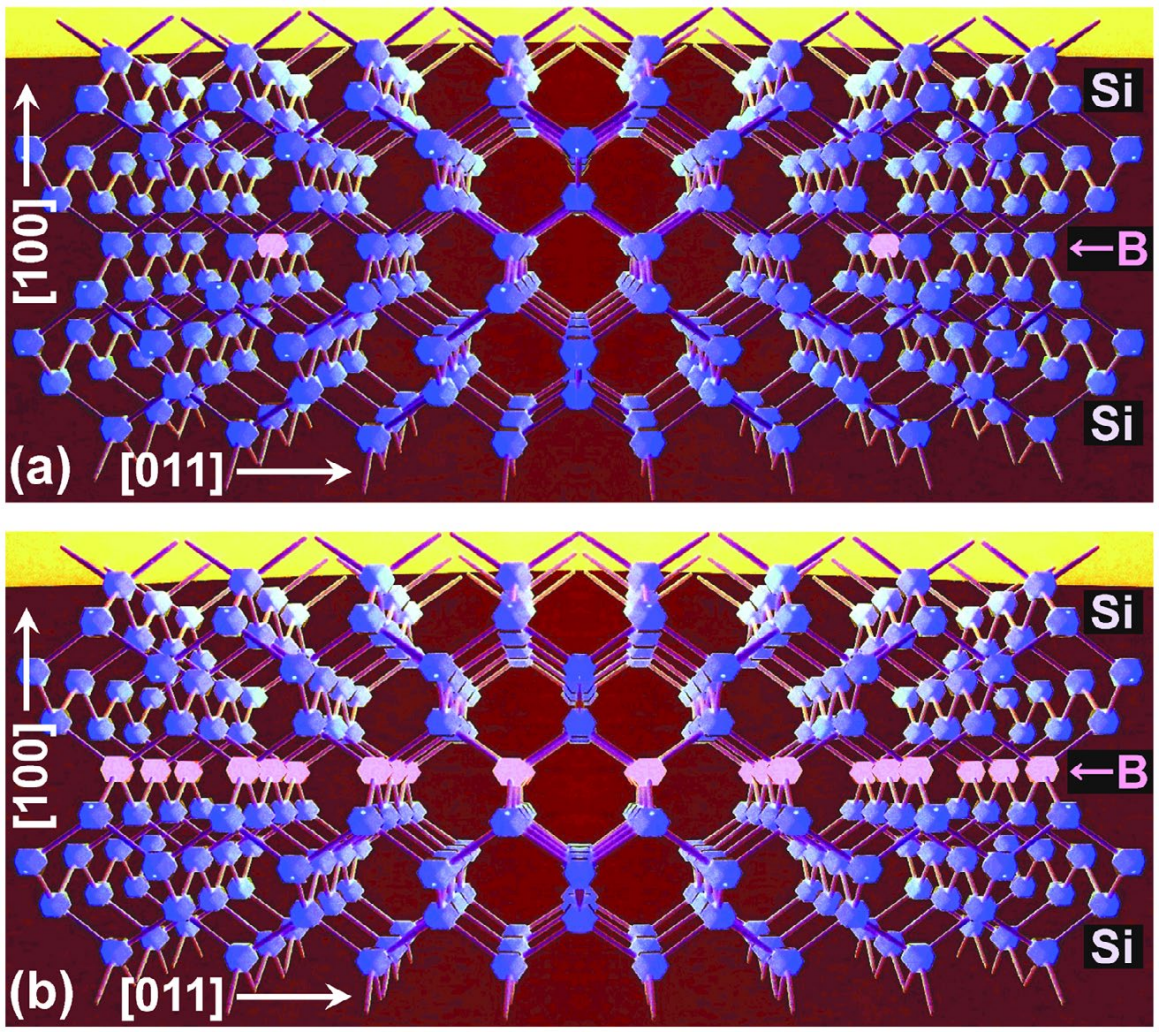
Cross-sectional views for ball-and-stick models of B-doped Si crystals with (a) conventional isolated-atom doping or (b) AL doping for a (100) plane. Blue and red balls are Si and B atoms, respectively. Sticks are covalent bonds for diamond structure.

**Figure 2.**
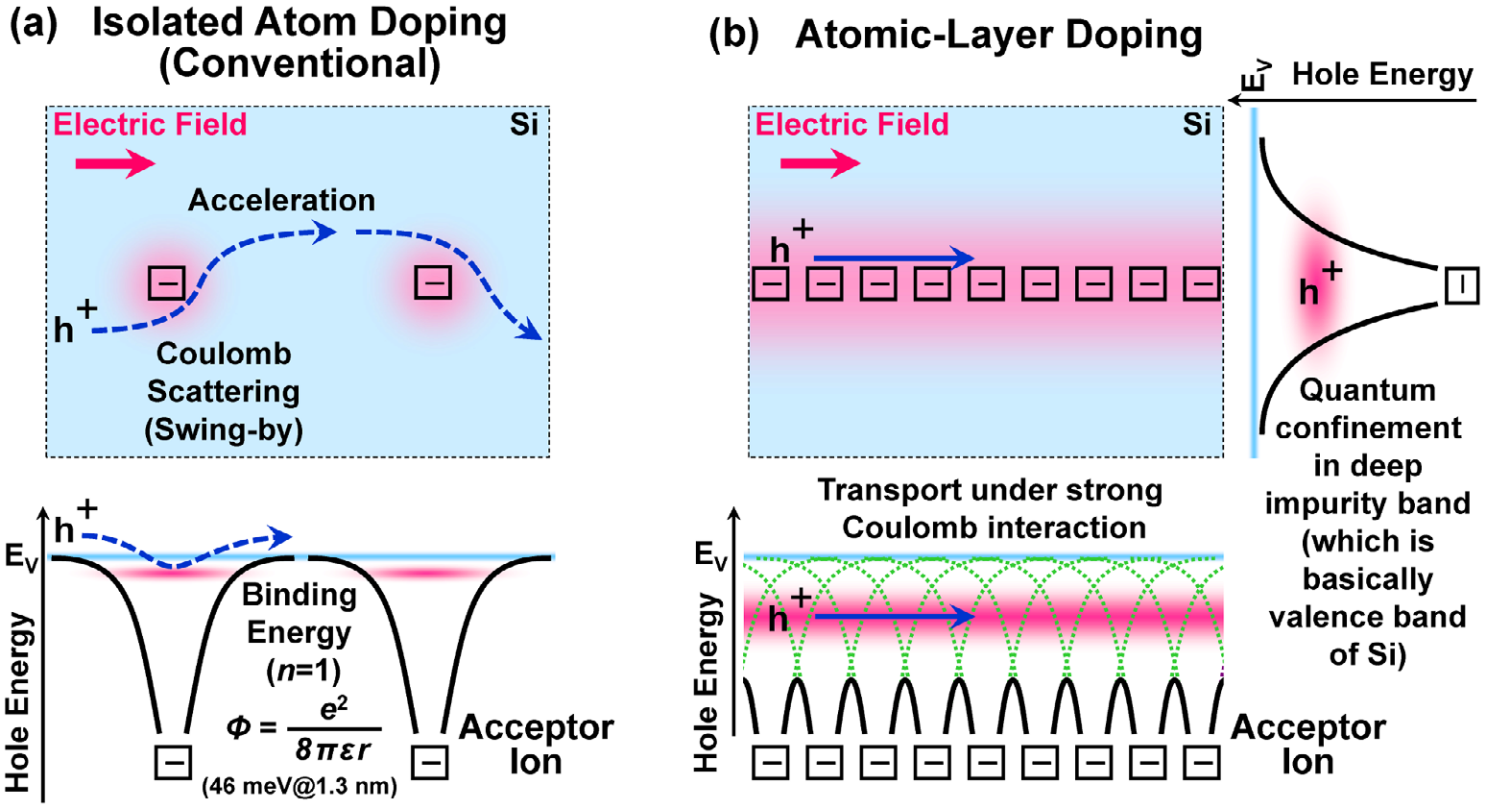
(Top) Plan-view images of carrier transport under lateral electric field in the cases with (a) scattering by isolated ions for the isolated-atom doping and (b) quantum confinement in 2-D ion sheet for the AL doping. Here, the carrier is a hole (‘h^+^’) in the valence band of Si crystal. (Bottom) Cross-sectional views of isolated or synthetic Coulomb potentials (*Φ*) affected by the B^-^ ions in the hole-energy-band diagrams.

An alternative to conventional isolated-atom doping (Figure 1(a)) is atomic-layer (AL) doping (or δ-doping) in epitaxy (Figure 1(b)). It has been investigated by use of molecular-beam epitaxy (MBE) and thermal CVD to realize low-resistive ultrathin semiconductor films as well as a two-dimensional (2-D) carrier transport system which will become important for next-generation nanoelectronic devices [14,31–45]. Here, from in-plane lattice constants, the sheet atom density of the 1 AL is calculated to be 6.78 × 10^14^ cm^−2^ and 6.67 × 10^14^ cm^−2^ respectively on an unstrained Si(100) and a 0.8%-tensile-strained Si(100). According to our knowledge based on historical semiconductor physics, transport of carriers in the valence band and the conduction band is limited by an ionized-impurity scattering due to Coulomb attractive (or repulsive) force between carriers and isolated-impurity ions in the case of isolated-atom doping (Figure 2(a)). In the case of AL doping, the carriers may be confined and, at the same time, transported in a 2-D Coulomb potential well (impurity band) which is synthesized by closely neighboring ionized impurity atoms (Figure 2(b)). Therefore, if the impurity atoms are highly condensed (as high as above 10^13^ cm^−2^ so that the atomic spacing for B atoms is close to the effective Bohr radius for a hole in an isolated-B-doped Si crystal [46]) and aligned in atomic-order thickness, there is a possibility that carrier transport and electronic transition differ from that of the isolated-atom doping. For example, such a spatial configuration is similar to a carrier transport system in an inversion layer of Si metal-oxide-semiconductor field-effect-transistor (MOSFET) with 2-D charged centers localized at SiO_2_/Si interface [47].

In our previous work on AL doping, to suppress thermal diffusion and to realize specific states far from thermal equilibrium, we performed B AL doping by plasma-induced Si epitaxy using B_2_H_6_ and SiH_4_ without substrate heating. Efficient B confinement within a ~2 nm-thick region in a Si cap layer was realized via low-energy plasma irradiation [48]. In this paper, detailed B depth profiles and behavior of carrier properties in the B AL-doped Si films are reported in order to explore their potential for semiconductor device applications.

## 2. Materials and methods

### 2.1. Substrate preparation and plasma-induced surface reactions without substrate heating

**Figure 3.**
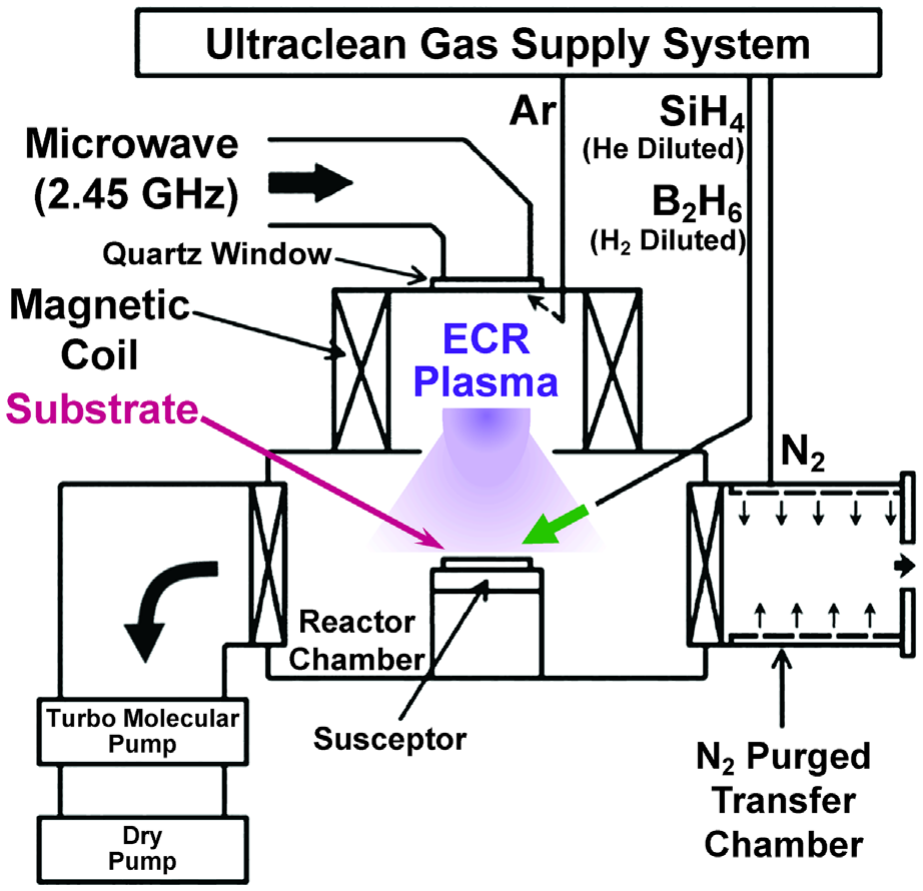
Schematic of our ECR Ar plasma-enhanced CVD system for the B AL-doped Si film formation.

By using our ECR Ar plasma-enhanced CVD (Figure 3) without substrate heating [21,22], B AL-doped Si films were formed on Si(100) through plasma-induced reactions of B_2_H_6_ and SiH_4_ under low-energy Ar plasma irradiation as follows. Substrates used were Si(100) wafers and they were cleaned for several cycles in a 4:1 solution of 96% H_2_SO_4_ and 30% H_2_O_2_ and treated in dilute hydrogen fluoride (DHF, a few percent hydrogen fluoride in water) solution to remove the native oxide and rinsed with deionized water just before loading into the reactor chamber. By the DHF treatment, it is well known that a Si atom on top of Si(100) is terminated by a few hydrogen atoms and the surface is effectively protected from native oxide formation at room temperature [49]. To minimize air contamination of the reactor chamber and to enhance desorption of water molecules physically adsorbed on the substrate, wafer loading was performed through a N_2_ purged transfer chamber combined with a gate valve. After wafer loading and evacuating, Ar gas was continuously supplied into a plasma-generation chamber at a pressure of 2.1 Pa. Then, B_2_H_6_ (1% in H_2_) was directly introduced into the reactor chamber. Subsequently, microwave (2.45 GHz, 100 W) was supplied through a quartz window on top of the plasma-generation chamber and electron-cyclotron resonance was induced at a specific magnetic field of 0.0875 T to form a B AL-adsorbed Si(100). Next, in the same manner, SiH_4_ (5% in He) was directly introduced into the reactor chamber, and microwave (2.45 GHz, 200 W) was supplied to deposit a Si cap layer on the B AL-adsorbed Si(100). This Si cap layer has an important role in electrically activating B atoms due to incorporation at the substitutional site in Si crystals, as well as avoiding undesirable surface effects, e.g. oxidation of B atoms, increase of contact resistivity with metal electrodes and carrier scattering by surface charged states. In this experiment, we set the as-deposited Si cap layer thickness at 7 nm and the thickness was finally reduced to 5 nm due to thermal oxidation during SiO_2_ CVD described in Section 2.3. Here, B_2_H_6_ and SiH_4_ partial pressures were 2 × 10^−4^ Pa and 3 × 10^−4^ Pa, respectively. To avoid plasma damage and intermixing at interfaces, a low-energy ECR Ar plasma condition with an Ar pressure as high as a few Pa is important to obtain peak ion energy as low as a few eV. For the B AL formation at 100 W, values of Ar^+^ flux density and peak ion energy were estimated to be about 2 × 10^15^ cm^−2^ s^−1^ and 0.3 eV, respectively. For the Si cap layer deposition at 200 W, values of Ar^+^ flux density and peak ion energy were estimated to be about 4 × 10^15^ cm^−2^ s^−1^ and 0.5 eV, respectively. Substrate temperature was suppressed below 100 °C during plasma exposure even for a few hundred seconds in the present condition at up to 200 W [21,22]. Here, it should be noted that *in situ* surface cleaning was not performed intentionally before the B AL formation. Metal contamination from stainless steel chambers was suppressed by quartz covers inside of the plasma-generation chamber and below the detection limit of our X-ray photoelectron spectroscopy measurement. Si incorporation rate from the quartz or deposit on it was at most 0.2 AL min^–1^ during the Ar plasma irradiation at 200 W and this corresponds to at most about 3% in the present Si deposition rate (about 1 nm min^–1^).

### 2.2. Material characterization

Crystallinity of Si cap layers deposited on the B AL-adsorbed Si(100) was *ex situ* evaluated by reflection high-energy electron diffraction (RHEED) in a high-vacuum electron beam irradiation system with an electron-acceleration voltage of 16 kV (electron wavelength 9.7 pm) after sample transfer within 5 min in cleanroom air. Deposited thickness was measured by atomic force microscope (AFM) after lift-off technique for thin film deposition on the partially SiO_2_-covered Si(100). Surface roughness for the as-deposited samples was also measured by AFM.

Initial B amount on top of the surface without Si cap layer deposition was evaluated *ex situ* by X-ray photoelectron spectroscopy (XPS) (Surface Science Laboratories (USA): SSX-100) with a take-off angle of 35° for photoelectrons. Peak intensities of photoelectrons from the core levels of Si 2*p* and B 1*s* were evaluated with a monochromatized X-ray of Al Kα line at 1487 eV without any surface treatment (such as ion beam etching). For the B amount (*N*_B_ [cm^−2^]), the calculations were performed by following equation: *N*_B_ = *N*_0_ ln{1 + *α I*_B_ (*I*_Si_ + *α I*_B_)^−1^}. Here, *α* is a relative sensitivity factor for B 1*s* (1.58; determined using a B-doped Si film standard)*, I*_B_ the photoelectron intensity for B 1*s*, *I*_Si_ the photoelectron intensity for Si 2*p*, and *N*_0_ a calibrated conversion factor (5.3 × 10^15^ cm^−2^).

Depth profile of B concentration and incorporated B amount (B dose) in the as-deposited B AL-doped Si films were measured by secondary-ion mass spectrometry (SIMS; FEI Company (USA) Atomika 4500) with ultralow primary ion (O_2_^+^) energy (500 eV) [50] and secondary-ion detection of Si^+^ and B^+^ at the Foundation for Promotion of Material Science and Technology of Japan (MST).

### 2.3. Electrical measurements

**Figure 4.**
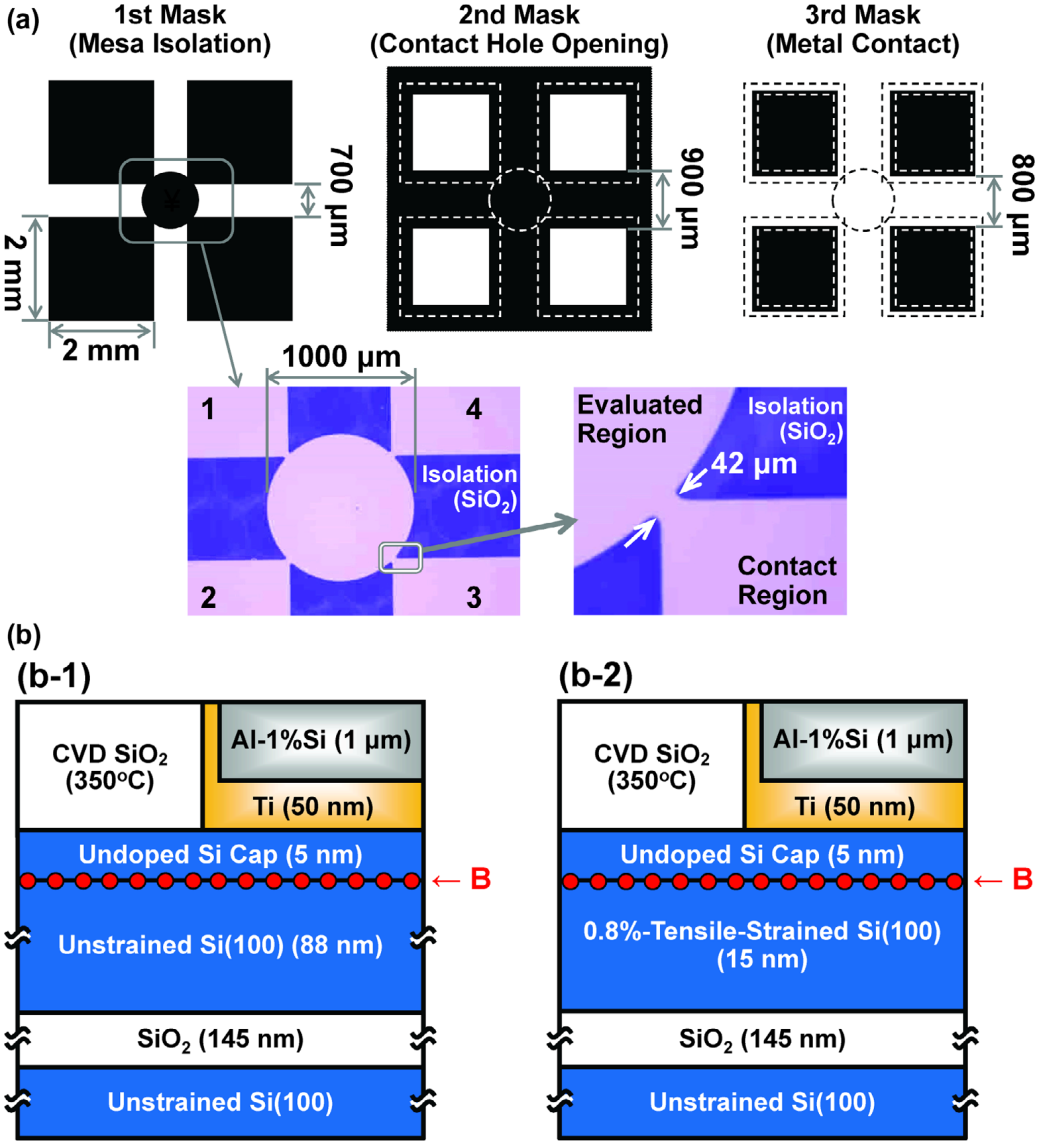
(a) Mask patterns for lithography and (b) schematic cross-sectional structures and typical film thicknesses near the edge of metal contact in the Hall-effect devices on (b-1) the unstrained SOI and (b-2) the 0.8%-tensile-strained SOI. Si cap layer thickness for the Hall-effect devices was fixed at 5 nm.

To determine the carrier type (*n* for electron or *p* for hole), carrier concentration and Hall mobility were evaluated at room temperature for the B AL-doped Si films via resistivity and Hall-effect measurements. Hall-effect measurements (magnetic flux of 0.6 T) were performed in the van der Pauw geometry [51–54], on a 1-mm diameter circular region in devices fabricated by SiO_2_ CVD, photo lithography, etching and metallization (Figure 4). The maximum process temperature was 350 °C for the SiO_2_ CVD for as-deposited B AL-doped Si films, and additional heat treatment was performed at 500–700 °C for 60 min in N_2_ atmosphere for other B AL-doped Si films after the SiO_2_ CVD. After passivation by second SiO_2_ CVD and contact-hole opening, DHF treatment for native oxide removal was performed and then metals (Ti and Al-1%Si) were sequentially deposited by radio-frequency Ar plasma sputtering with the metal targets without substrate heating. Here, it should be noted that *in situ* surface cleaning for the Si cap layer was not performed intentionally before the metal deposition.

Especially for the electrical measurements, an unstrained Si(100)-on-insulator (SOI) substrate (Shin-Etsu Handotai, Japan) and a 0.8%-tensile-strained SOI substrate (Soitec, France) were used to reduce parallel conduction in the substrate material. The circular region was defined simply by etching of SOI layer. The purpose of SiO_2_ CVD is to protect surface of the Si cap layer and define the areas of contact holes. Here, the unstrained SOI substrate has an 88-nm-thick *p*-type Si(100) with resistivity of 9–18 Ω cm (sheet resistivity 1–2 MΩ/square, carrier concentration < 10^15^ cm^−3^) as a top Si layer and a 145-nm-thick SiO_2_ as a buried oxide layer on a thick *p*-type Si(100) substrate. The strained SOI substrate has a 15-nm-thick unintentionally-doped Si(100) as a top Si layer.

## 3. Results and discussion

### 3.1. Influence of B AL and strain upon Si cap layer deposition

**Figure 5.**
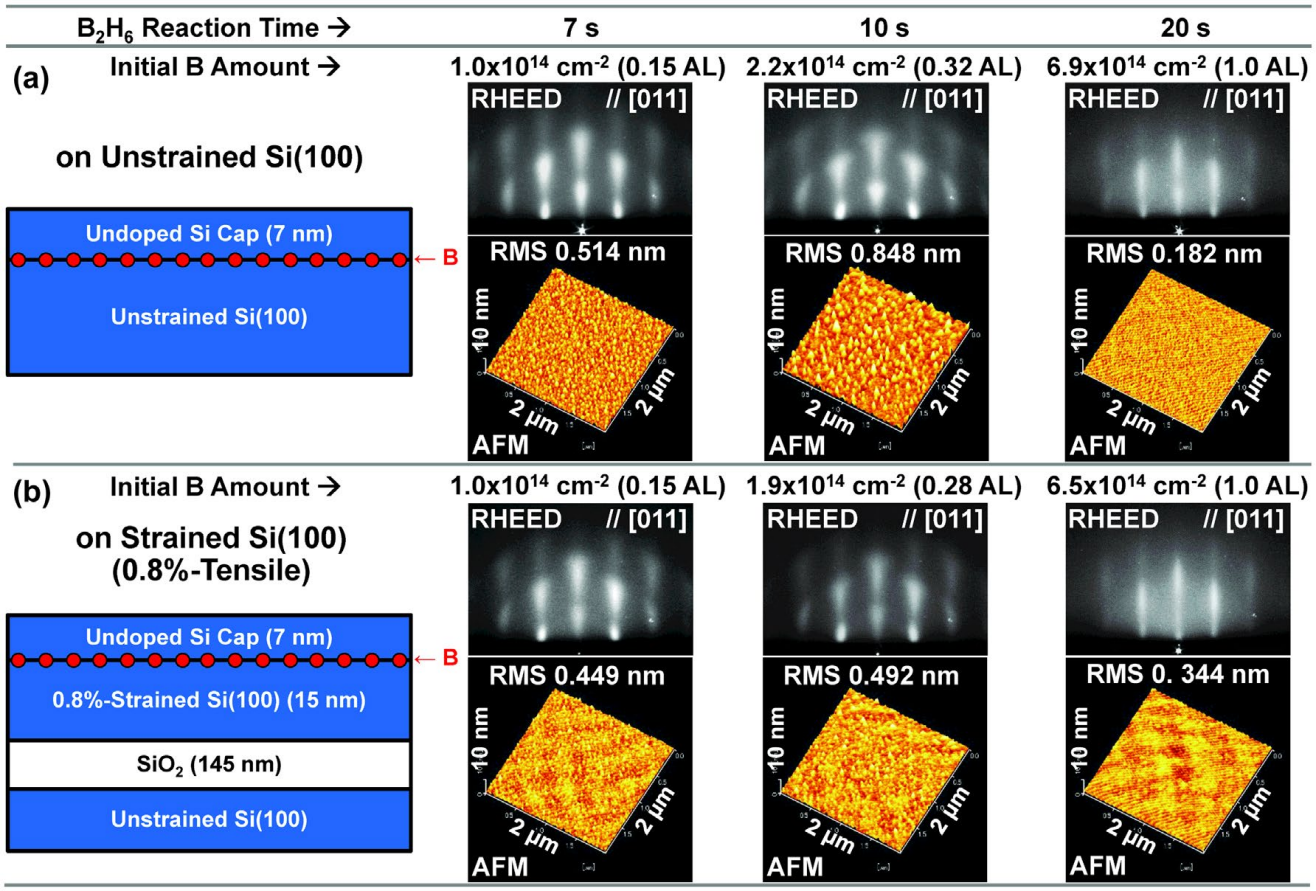
B_2_H_6_ reaction time dependence of initial B amount, RHEED patterns and AFM images for the B AL-doped Si films epitaxially grown on (a) the unstrained SOI and (b) the 0.8%-tensile-strained SOI. Root-mean-square (RMS) values of surface roughness are also shown in the AFM images. Si cap layer thickness was fixed at 7 nm.

By the B AL formation on Si(100), Si cap layer deposition was influenced as follows. First, in the case of larger initial B amount than 1 AL, a halo pattern was observed in the RHEED pattern; this indicated that crystallinity was lost and the Si cap layer becomes amorphous. On the other hand, in the case of initial B amount up to 1 AL, streaks or streaky spots are observed in the RHEED pattern with a slightly rough surface shown in the AFM image (Figure 5). Therefore, it is concluded that the Si cap layer can be epitaxially grown on the B AL-adsorbed Si(100) up to 1 AL, and excess B to 1 AL causes crystallinity degradation in the Si cap layer. Moreover, especially in the case of initial B amount just at 1 AL, continuous (undotted) streaks are observed in the RHEED pattern with a smoother surface in the AFM image. This implies that 1 AL might be some kind of critical point, and lowering the B surface coverage from 1 AL induces surface roughening. This seems to be related to surface conformality in atomic scale. Such a phenomenon is quite interesting and this seems to be important for high quality and ultrathin B AL-doped Si film formation, while its mechanism has not yet been clarified. Additionally, because a saturation manner in the B AL formation at an atomic-order thickness was not observed [47], the surface reaction of B_2_H_6_ on Si(100) seems not to be self-limited. Therefore, we could not confirm suppression of the B-cluster (B-B bond) formation in this experiment. However, from the viewpoint of the critical point of 1 AL as a magic number, there still exists a possibility that almost ideal B AL is formed with suppressed B-cluster (B-B bond) formation. Moreover, because the RMS values of surface roughness are well suppressed at most below 1 nm and much smaller than the Si cap layer thickness, it is expected that most of the B atoms are buried under the Si cap layer, if B segregation on top of growing surface is suppressed, as discussed in the next section.

### 3.2. SIMS depth profile of B concentration

**Figure 6.**
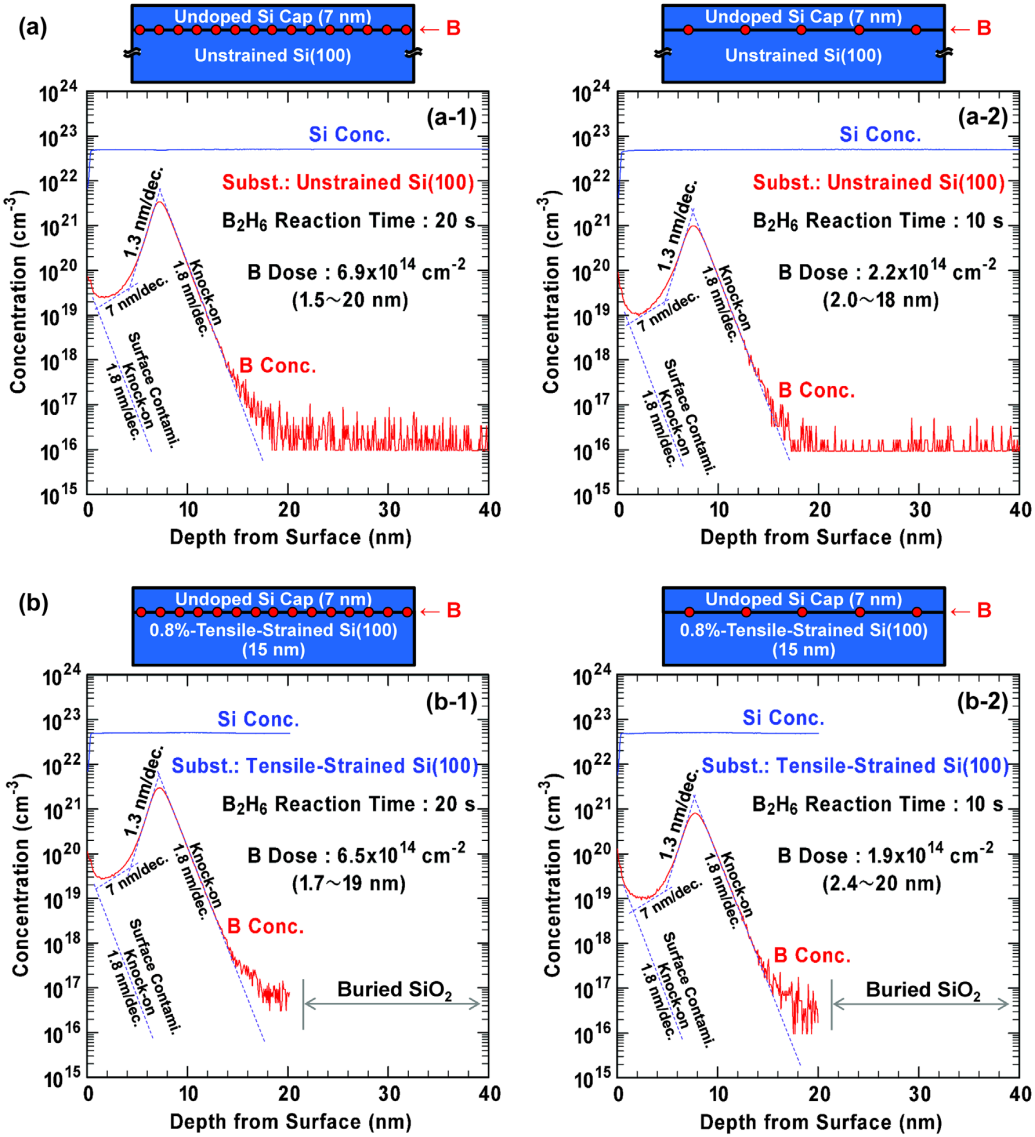
SIMS depth profiles of B and Si concentrations in as-deposited B AL-doped Si films epitaxially grown on (a) unstrained SOI and (b) 0.8%-tensile-strained SOI. B_2_H_6_ reaction time was (a-1, b-1) 20 s and (a-2, b-2) 10 s. Si cap layer thickness was fixed at 7 nm. Here, it is confirmed that the B doses, which are integrated values of the B concentration in the regions shown in the figures, are in good agreement with the initial B amounts.

B segregation is a phenomenon observed in Si epitaxial growth at relatively high temperatures [40]. During the B segregation, some segregated B atoms are incorporated in the Si cap layer and the amount tends to be decreased gradually. As a result, B concentration peak in the Si cap layer becomes broader and atomic-order confinement becomes difficult. Therefore, it is suggested that lowering the Si growth temperature is key for ideal AL doping, although there exists a trade-off between the B confinement and epitaxial quality in the case of MBE and thermal CVD at relatively high temperature with substrate heating. In order to explore the effectiveness of our plasma-induced Si epitaxial growth technique without substrate heating, depth profiling of B concentration in Si was performed by SIMS for the B AL-doped Si epitaxial films on Si(100) (Figure 6). In the epitaxial Si cap layers on B AL-adsorbed Si(100) with initial B amount of 2 × 10^14^ cm^−2^ or 7 × 10^14^ cm^−2^ (about 0.3 AL or 1 AL), the depth profiles show a clear decay slope as steep as 1.3 nm/decade at the depth of 5–7 nm (near the B AL at initial Si(100)). The slope is considered to be determined mainly by surface roughness generated during etching cycles in the SIMS measurements [50]. Decay characteristics with a slope of 1.8 nm/decade is also observed at the depth of 8–14 nm and this can be understood as a knock-on effect typically observed in SIMS due to the existence of B AL. As discussed later in Section 3.4, it is considered that, at lower temperatures below 400 °C, thermal diffusion of B is significantly suppressed and realistic B concentration in the Si substrate region beneath the initial B AL is negligibly small. Because a pile-up near the surface seems to be independent of the initial B amount, an additional knock-on effect due to contaminants adsorbed at the surface is considered and the slope of 1.8 nm/decade can also be applicable to the depth of 1–4 nm. Additionally, it is clear that the minimum B concentration level in the Si cap layer increases with the initial B amount. From these characteristics, one more additional component in the role-up can be extracted at the depth of around 3–4 nm and the slope is estimated to be around 7 nm/decade (as shown by dotted lines in Figure 6). This fact implies that the role-up is derived from B segregation from the B AL, although the amount of the segregated B atoms is much smaller than the local B dose at the depth around 5–7 nm. From these results, it is suggested that the plasma contributes not only to hydrogen desorption and Si epitaxial growth but also to site exchange between Si and B at the growing surface in the present plasma conditions. If we want to obtain a highly pure Si cap layer on the B AL-adsorbed Si(100), the B segregation has to be suppressed more effectively. Therefore, exploring lower microwave power conditions for plasma generation with keeping crystallinity might be valuable as an issue for ideal AL doping. Moreover, it should be noted that the effect of the 0.8%-tensile strain upon the B concentration profiles is negligibly small in our experiments without a slight reduction of total B dose.

In the case of thermal CVD at 550 °C, compared to the B doping, it has been reported that P segregation in Si epitaxial growth is more significant [55]. If we try to grow a P AL-doped Si film with suppressed P segregation, our ECR Ar plasma-enhanced CVD might also be expected to be useful due to elimination of substrate heating. However, as reported above, further investigations based on experiments will be necessary to achieve an ideal AL-doped structure.

### 3.3. Carrier properties

**Figure 7.**
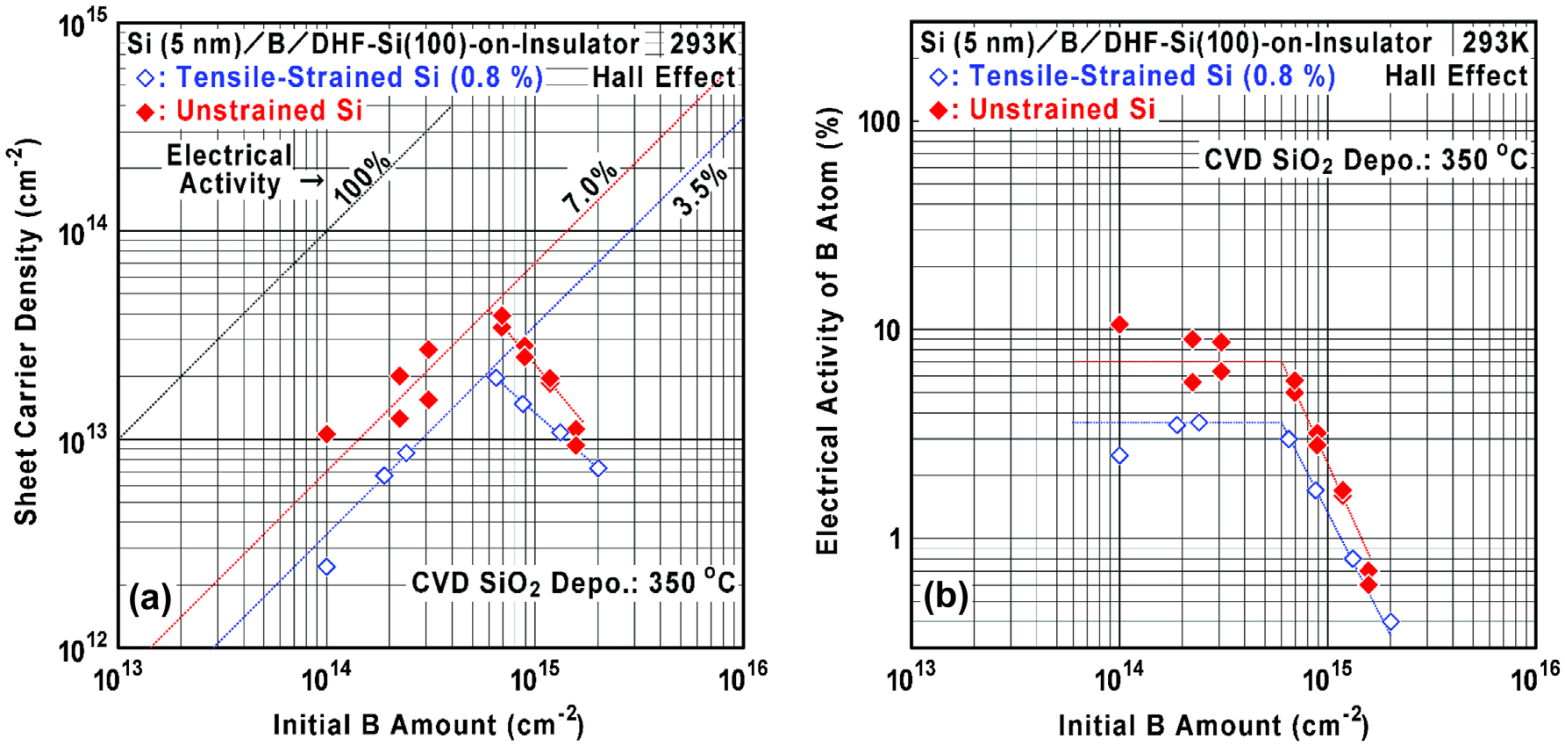
Initial B amount dependence of (a) sheet carrier density and (b) electrical activity of B atom in the B AL-doped Si films on the unstrained SOI (filled marks) and the 0.8%-tensile-strained SOI (open marks). The electrical activity was calculated using the data shown in (a).

Figure 7 shows initial B amount dependence of the sheet carrier density (*n*_s_) and electrical activity of B atom in the B AL-doped Si films on the unstrained SOI and the 0.8%-tensile-strained SOI. After the Hall-effect device fabrication (maximum process temperature 350 °C), it is found that the sheet carrier density tends to increase with the initial B amount up to around 7 × 10^14^ cm^−2^ (about 1 AL) and then the density tends to decrease. As a result, maximum sheet carrier densities as high as 4 × 10^13^ cm^−2^ and 2 × 10^13^ cm^−2^ (electrical activity of about 7% and 3.5%) were achieved respectively for the unstrained SOI and 0.8%-tensile-strained SOI. As shown in Figure 7(b), electrical activity is drastically reduced when the initial B amount exceeds 1 AL. Absolute values of sheet carrier density and electrical activity become lower by existence of the 0.8%-tensile strain, while the tendency is almost the same. Here, it is important to consider a contribution of B segregation to carrier generation. As discussed in Section 3.2, the segregated B atoms are incorporated at the depth of 1–4 nm and the amount is estimated to be at most around 9 × 10^12^ cm^−2^ (a product of 3 × 10^19^ cm^−3^ and 3 × 10^−7^ cm) and 5 × 10^12^ cm^−2^ (a product of 1.5 × 10^19^ cm^−3^ and 3 × 10^−7^ cm) respectively for the unstrained SOI and 0.8%-tensile-strained SOI. The segregated B amount is apparently much smaller than the peak sheet carrier density shown in Figure 7(a). Therefore, it is suggested that the carriers and ionized B atoms dominantly exist at the initial B AL on Si(100) for the initial B amount of around 1 AL, which is similar to the situation shown in Figures 1(b) and 2(b).

In the case of B AL doping by thermal CVD, it has been reported that the B AL without Si cap layer tends to be deactivated at 500 °C and atomic-order Si cap layer deposition on the B AL at 180–300 °C is effective to improve the activation before heat-up to 500 °C for Si cap layer deposition [44,45]. This implies that a small amount of Si atoms on the B AL partly suppresses the formation of B-clusters at 500 °C. As a result, by using thermal CVD, a sheet carrier density as high as 1.7 × 10^13^ cm^−2^ was achieved at the initial B amounts of 2.0 × 10^14^ cm^−2^ and 7.0 × 10^14^ cm^−2^. Compared to this value (1.7 × 10^13^ cm^−2^), it should be noted that a much larger value (4 × 10^13^ cm^−2^) can be obtained by our ECR Ar plasma-enhanced CVD. At present, this seems reasonable based on the effectiveness of lowering temperature for Si cap layer deposition. In order to overcome the present limitation of electrical activation in the B AL doping, further investigations will be carried out.

**Figure 8.**
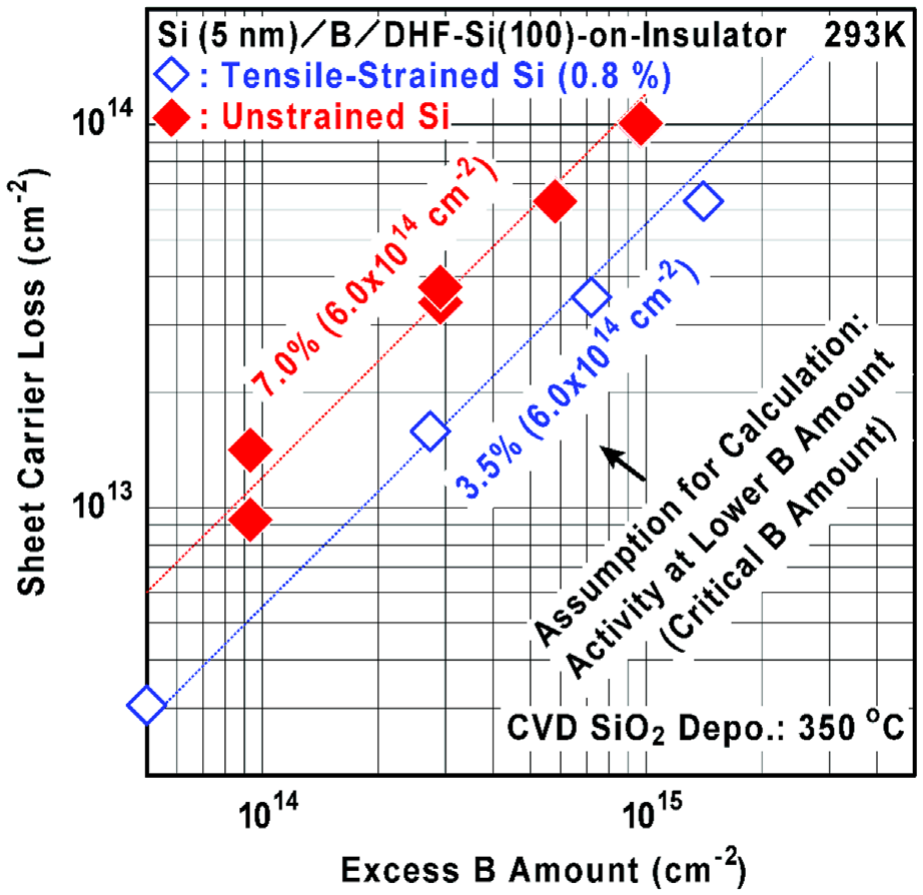
Relationships between excess B amount and sheet carrier loss calculated using the data shown in Figure 7(a) when a specific electrical activity at lower B amount (7% and 3.5%) and a specific critical B amount (6.0 × 10^14^ cm^−2^) are assumed.

Figure 8 shows the relationship between excess B amount and sheet carrier loss calculated using the data shown in Figure 7(a) when a specific electrical activity (7% or 3.5%) at the lower B amount and a specific critical B amount (6.0 × 10^14^ cm^−2^) are assumed. Here, excess B amount is defined as an increase of the initial B amount from the critical one. Sheet carrier loss is defined as a decrease of the sheet carrier density from the maximum at the critical B amount. It is found that the sheet carrier loss is almost proportional to the excess B amount. It should be noted that many of the incorporated B atoms are inactive and do not contribute to carrier generation. Therefore, there seem to be two dominant origins of the B deactivation, respectively, for the lower B amount below 1 AL and for the larger B amount above 1 AL. For example, the former is existence of residual B-H bonds and the latter is B-cluster (B-B bond) formation as well as crystallinity degradation. Hence we have to consider reduction of the residual B-H bonds to enhance the formation of Si-B bonds, especially in the initial stage of Si cap layer deposition.

**Figure 9.**
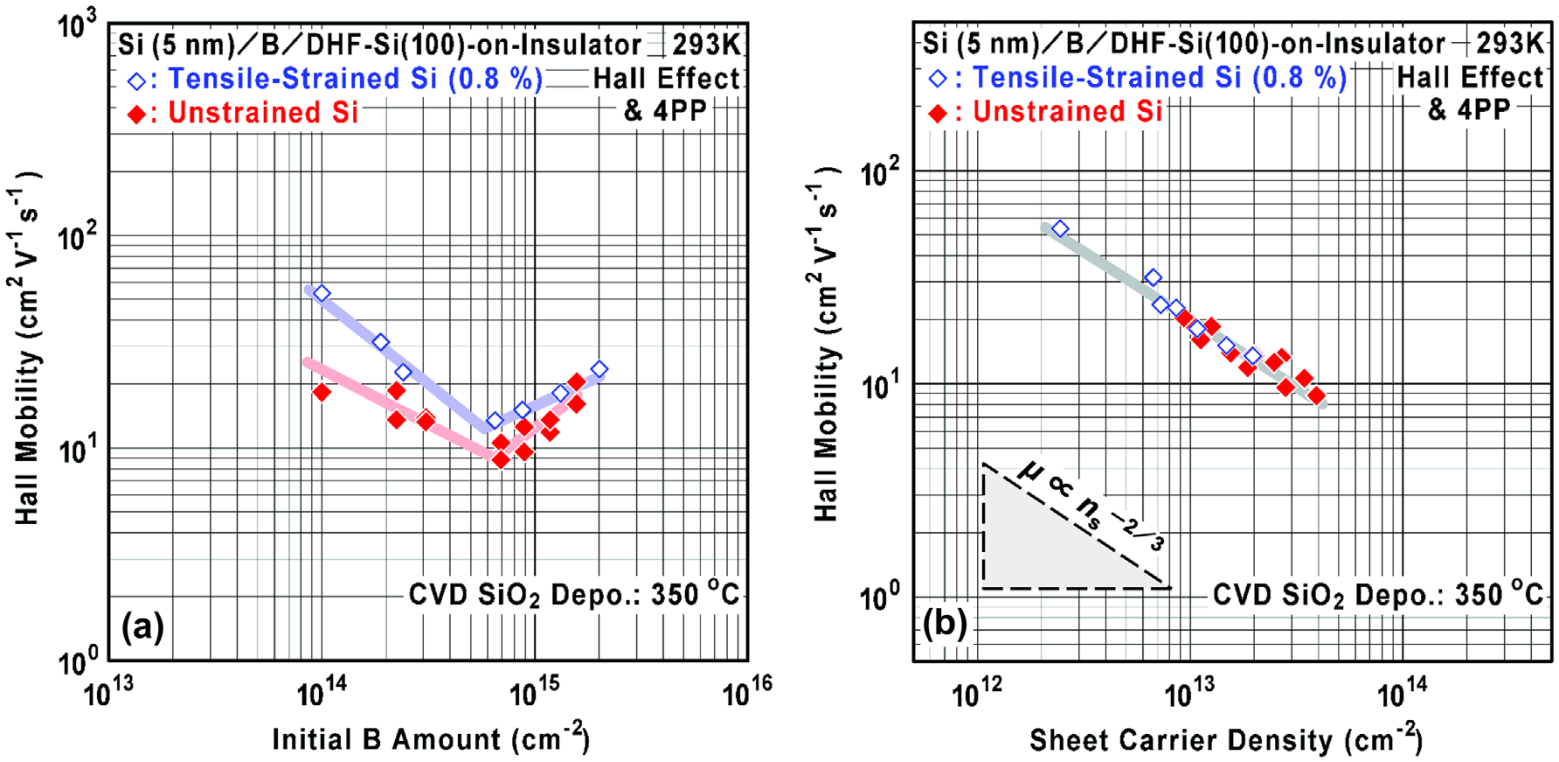
Dependence of Hall mobility (*μ*) on (a) initial B amount and (b) sheet carrier density (*n*_s_) in the B AL-doped Si films on the unstrained SOI (filled marks) and the 0.8%-tensile-strained SOI (open marks).

Figure 9 shows dependence of Hall mobility (*μ*) on the initial B amount and sheet carrier density. It is found that, with increasing initial B amount, the mobility tends to decrease and then increase when the B amount is larger than 7 × 10^14^ cm^−2^ (Figure 9(a)). It is also found that Hall mobility depends on the tensile strain in the SOI. For the maximum sheet carrier density, a minimum Hall mobility around 10–13 cm^2^ V^−1^ s^−1^ was observed. The mobility increase at larger B amounts is attributed to the reduction of ionized B atom density and we have to take into account the fact that electrical activation was apparently different between the cases for the unstrained SOI and for 0.8%-tensile-strained SOI, as shown in Figure 7. By replotting the mobility data for sheet carrier density (Figure 9(b)), it is clarified that all the data are located on a single line whose slope is proportional to *n*_s_^−2/3^. This indicates that the mobility is dominantly determined by the sheet density of ionized B atoms, and the effect of the 0.8%-tensile strain upon mobility is negligibly small. Because the mobility value is in the same level or slightly lower than a reported value of conventional heavily B-doped Si (around 20 cm^2^ V^−1^ s^−1^ or lower for above 10^21^ cm^−3^ [56]), there is a possibility that carrier concentration has reached a specific level for degenerate semiconductor with an impurity-band formation [57]. Moreover, although a hole mobility in Si is generally enhanced by tensile strain [58], such an enhancement is scarcely observed in our B AL-doped Si. From this fact, it is considered that the mobility is strongly influenced by quantum confinement in the synthetic Coulomb potential well (with strong Coulomb interaction), as shown in Figure 2(b). In other words, the mobility must be for a special situation of 2-D in-plane transport in which out-of-plane transport perpendicular to the surface is strongly limited. Takagi et al. [47] reported that inversion layer mobility in Si MOSFET is enhanced by screening effect of free carriers and degraded by Coulomb scattering at 2-D charged centers localized at the SiO_2_/Si interface[Bibr CIT0047]. From the viewpoint of carrier scattering phenomenon by 2-D charges, their situation is similar to ours, while a combination of charge types is different such as repulsive force or attractive force and order of sheet charge density is largely different. Because their mobility degradation tendency by the Coulomb scattering (*μ* ∝ *N*_int_^−*γ*^; *γ* ≤ 1, *N*_int_: sheet density of the 2-D interface charged centers) is similar to our result (*μ* ∝ *n*_s_^−2/3^), hole mobility in our case might also be determined in a trade-off relation between the screening effect by the 2-D free carriers and the Coulomb scattering effect by the 2-D charged centers (ionized B atoms). Here, it should be noted that *n*_s_ is always equal to *N*_int_ in our case and we have to compare them carefully based on the difference to the case of inversion layer in Si MOSFET. From the viewpoint of semiconductor device application, for example, introduction of such AL doping in the Esaki-tunnel diode is expected to increase a local built-in potential at a *p*-*n* junction and to contribute to enhancement and uniformity of interband-tunnel current [59,60].

### 3.4. Heat-treatment effect on carrier properties

**Figure 10.**
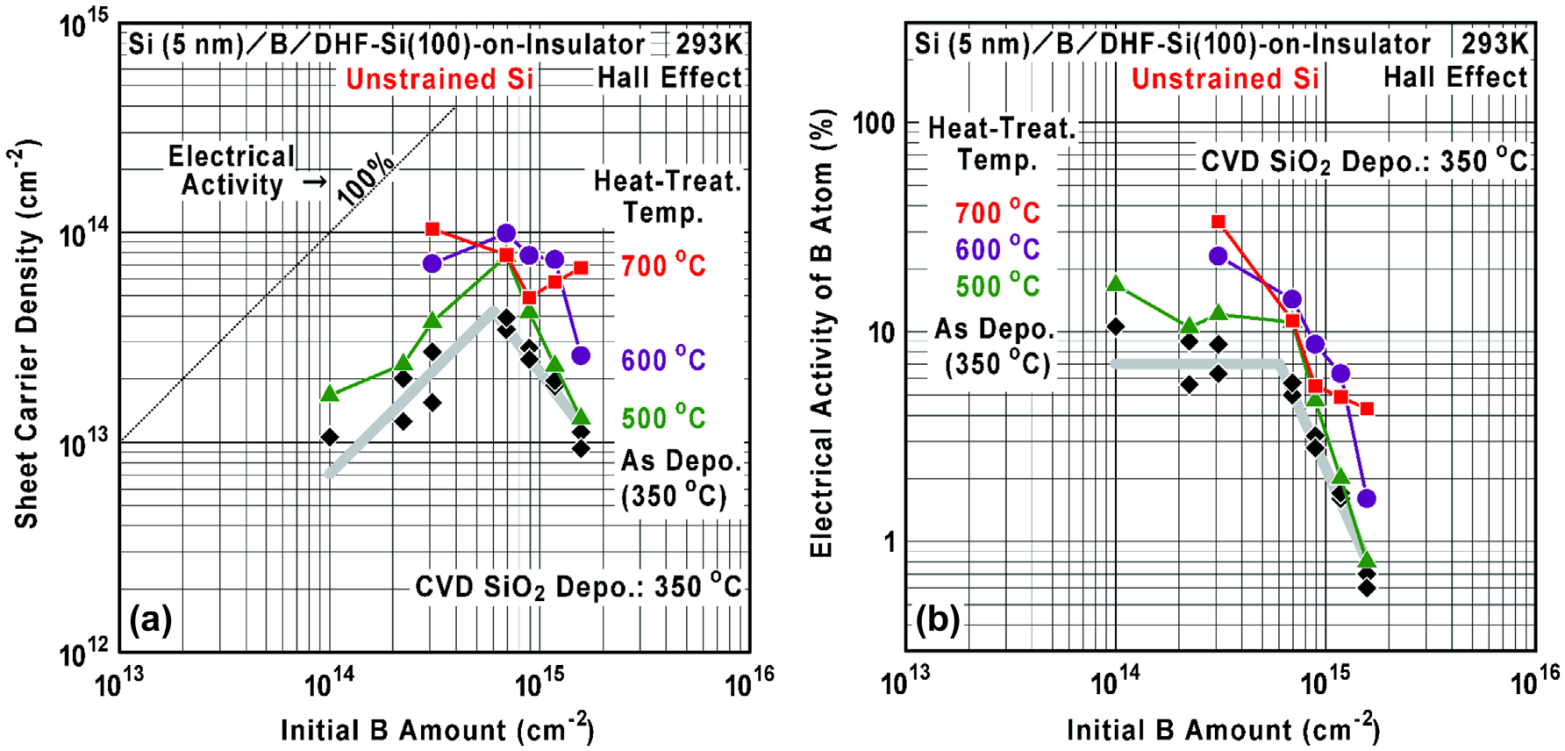
Initial B amount dependence of (a) sheet carrier density and (b) electrical activity of B atom in the B AL-doped Si films on the unstrained SOI at various heat-treatment temperatures for 60 min. The electrical activity was calculated using the data shown in (a).

**Figure 11.**
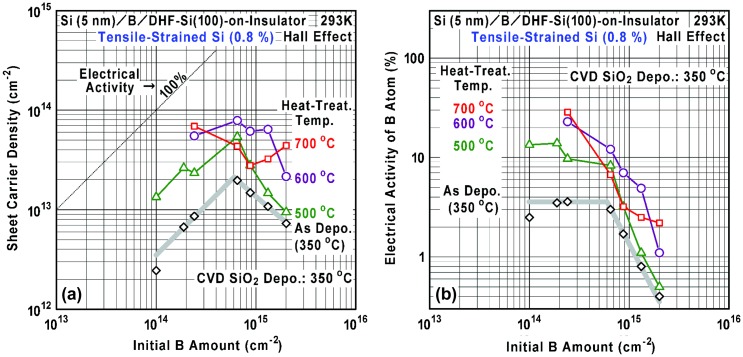
Initial B amount dependence of (a) sheet carrier density and (b) electrical activity of B atom in the B AL-doped Si films on the 0.8%-tensile-strained SOI at various heat-treatment temperatures for 60 min. The electrical activity was calculated using the data shown in (a).

Figures 10 and 11 show initial B amount dependences of the sheet carrier density and electrical activity of B atom at various heat-treatment temperatures for 60 min. It is found that the sheet carrier density tends to be increased with the temperature up to 600 °C. As discussed in Section 3.3, an important origin of B activation is considered to be hydrogen desorption from the residual B-H bonds and Si-B bond formation enhanced by the heat treatment. Another origin of activation is considered to be decrease of local B concentration due to thermal diffusion rather than the B-cluster formation. For example in the as-deposited B AL-doped Si films, if most ionized B atoms (4 × 10^13^ cm^−2^) are confined in a 3 AL-thick Si layer, average carrier concentration reaches as high as 10^21^ cm^−3^ and this can be attributed to the degenerate semiconductor [54] with the conventional isolated-atom doping. In this way, due to atomic-order localization of B-doped region in Si with as high electrical activity as 7%, 2-D impurity-band formation is expected even for the as-deposited B AL-doped Si films under suppressed thermal diffusion. Here, such high average carrier concentration is much larger than that at the reported solubility limit of B in bulk Si at thermal equilibrium (typically about 10^20^ cm^−3^ at 1000 °C) [61,62]. Formation of some silicon borides (e.g. SiB_3_ and SiB_6_) has been also found under B supersaturation condition at high temperatures [63]. Additionally, compared to the value (1.7 × 10^13^ cm^−2^) obtained by thermal CVD, a much larger value (8 × 10^13^–1 × 10^14^ cm^−2^) can be obtained by heat treatment at 500–600 °C after our ECR Ar plasma-enhanced CVD. This also strongly supports the effectiveness of lowering temperature of Si cap layer deposition for improvement of B activation. Therefore, B AL doping in Si by our ECR Ar plasma-enhanced CVD technique can be applicable effectively for important parts of various Si-based semiconductor devices, e.g. a low-resistive metal/semiconductor contact [64,65], a *p*-*n* junction [25,29] and an Esaki-tunnel junction [1,2,59,60] utilizing local built-in-potential control by heavy doping with high carrier concentration far above 10^19^ cm^−3^. Comparing the cases at 600 °C and 700 °C, a few cross-over points (coexistence of deactivation and activation tendency) are observed and the situation seems to be complicated. It is considered that there exists a competition among B-cluster formation (deactivation) and normal/enhanced B diffusion (activation) [66], which are sensitively dependent on the B concentration especially at 700 °C.

**Figure 12.**
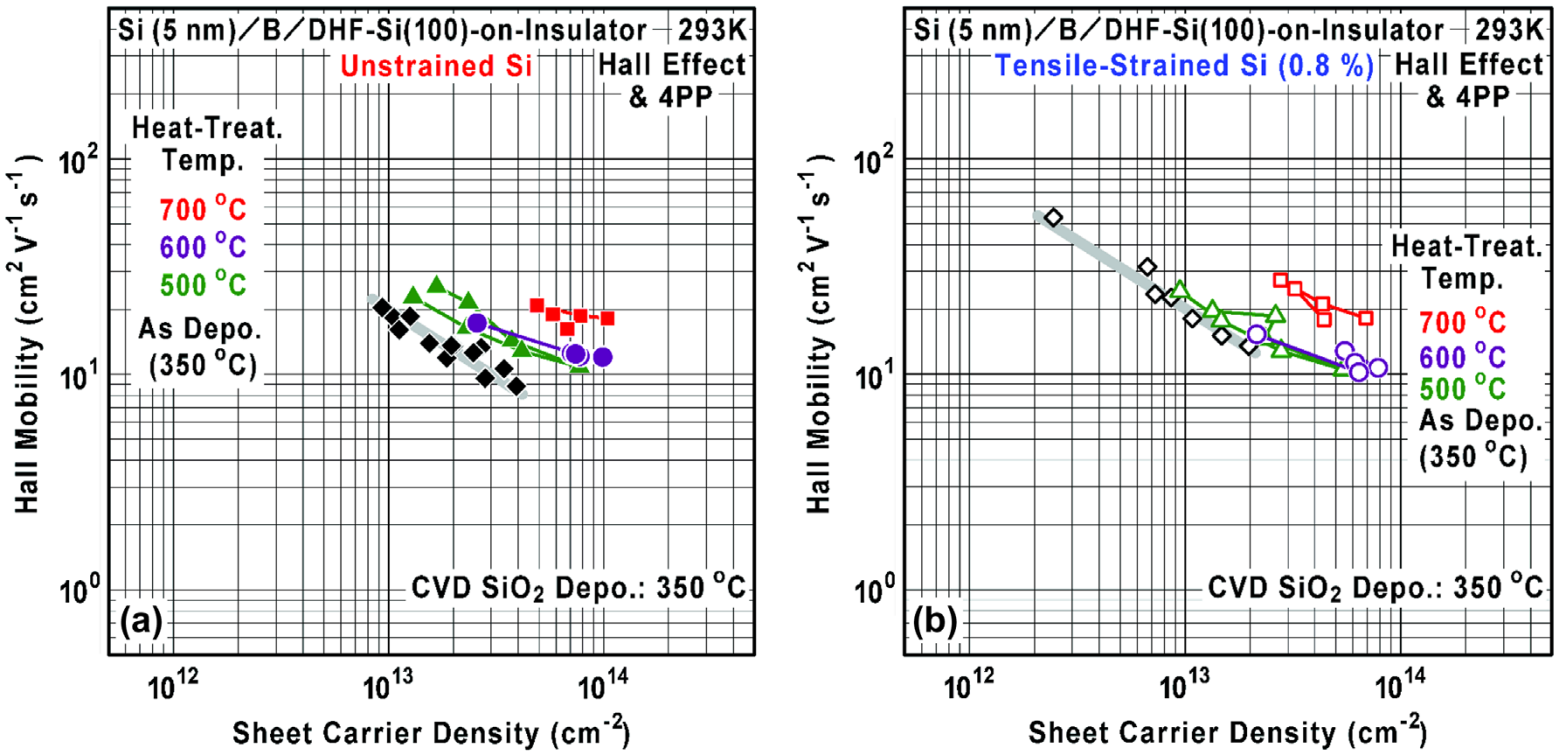
Relationship between sheet carrier density and Hall mobility in the B AL-doped Si films on (a) the unstrained SOI (filled marks) and (b) the 0.8%-tensile-strained SOI (open marks) for various heat-treatment temperatures.

Figure 12 shows the relationship between the sheet carrier density and Hall mobility for various heat-treatment temperatures. By the heat treatment at 500–600 °C, it is found that the mobility curves tend to shift to the right-hand direction, i.e. the mobility is scarcely changed even when the sheet carrier (ionized B atom) density is largely increased. On the other hand, by the heat treatment at 700 °C, both the sheet carrier (ionized B atom) density and mobility increase. From this result, decrease of local B concentration due to thermal diffusion and/or crystallinity improvement are considered dominantly as an origin of activation. Moreover, because thermal diffusion causes widening of the 2-D Coulomb potential well (impurity band) thickness, contribution of quantum confinement to the mobility will be modulated as typically reported for δ-doping of Si in MBE-grown GaAs [32]. Here, De Salvador et al. [67] have reported diffusivity of ^11^B spike in the ^10^B-doped Si prepared by MBE, ion implantation and low-temperature recrystallization. From their diffusivity (*D*) and heat-treatment time (*t*), approximate order of diffusion length, *L* ≡ (*D t*)^1/2^, perpendicular to the initial B AL can be estimated to have a temperature dependence as 0.6, 3.3, and 10 nm at 500, 600, and 700 °C, respectively, at ^10^B concentration of 2.8 × 10^19^ cm^−3^. If such dependence is applied to our case, it can be understood that the lowering of local ionized B concentration and the mobility enhancement proceed by the thermal diffusion even for higher electrical activation. Furthermore, by extrapolation from the above diffusion-length dependence, it is also considered that, at lower temperatures below 400 °C, thermal diffusion of B is significantly suppressed and realistic B concentration in the Si substrate region beneath the initial B AL is negligibly small. Such estimation needs to be considered carefully, because their maximum B concentration was at most 2.8 × 10^19^ cm^−3^, which was far below ours (> 10^21^ cm^−3^), and there is a possibility that the absolute value of *L* might be modulated largely. Additionally, the B cluster formation is expected to suppress a part of the B diffusion. Therefore, the heat-treatment temperature dependence of mobility we have observed is considered to be a complicated result of the combination of these effects. Finally, it is also found that the 0.8%-tensile strain seems to suppress the shift of mobility curves especially at 500–600 °C. This might be caused by alternation of thermal diffusion characteristics and/or crystallinity improvement due to the strain, while the enhancement of B activation at 500–600 °C seems to be scarcely dependent on the strain (Figures 10(a) and 11(a)).

## 4. Conclusions

B concentration depth profiles and carrier properties were evaluated for the B AL-doped Si films with suppressed thermal diffusion. Typically for the epitaxial Si cap layers on the B AL-adsorbed Si(100) with initial B amount of 2 × 10^14^ cm^−2^ and 7 × 10^14^ cm^−2^ (about 0.3 AL and 1 AL), SIMS depth profile of B concentration showed a clear decay slope as steep as 1.3 nm/decade. After Hall-effect device fabrication (maximum process temperature 350 °C), the dominant carrier was a hole and the maximum sheet carrier densities as high as 4 × 10^13^ cm^−2^ and 2 × 10^13^ cm^−2^ (electrical activity ratio of about 7% and 3.5%) were measured respectively for unstrained Si and 0.8%-tensile-strained Si with Hall mobility around 10–13 cm^2^ V^−1^ s^−1^. Moreover, mobility degradation was not observed even when sheet carrier (ionized B) density was largely increased by heat treatment at 500–700 °C. There is a possibility that the local carrier (ionized B atom) concentration around the B AL in Si reaches around 10^21^ cm^−3^, and 2-D impurity-band formation and strong Coulomb interaction is expected under suppressed thermal diffusion. The behavior of carrier properties for heat treatment at 500–700 °C implies that thermal diffusion causes broadening of the B AL in Si and decrease of local B concentration.

## Disclosure statement

No potential conflict of interest was reported by the authors.

## Funding

This study was partially supported by a Grant-in-Aid for Scientific Research on Priority Areas [number 18063001] from the Ministry of Education, Culture, Sports, Science and Technology of Japan, the Cooperative Research Project Program of the Research Institute of Electrical Communication, Tohoku University and the Japan Society for the Promotion of Science (JSPS) Core-to-Core Program, A. Advanced Research Networks ‘International Collaborative Research Center on Atomically Controlled Processing for Ultralarge Scale Integration’.
